# Blood-based bioenergetic profiling is related to differences in brain morphology in African Americans with Type 2 diabetes

**DOI:** 10.1042/CS20180690

**Published:** 2018-12-05

**Authors:** Gargi Mahapatra, S. Carrie Smith, Timothy M. Hughes, Benjamin Wagner, Joseph A. Maldjian, Barry I. Freedman, Anthony J. A. Molina

**Affiliations:** a. Department of Internal Medicine, Section on Gerontology and Geriatric Medicine, Wake Forest University Health Sciences, Winston-Salem, NC, 27157, USA; b. Centers for Genomics and Personalized Medicine Research and Diabetes Research, Wake Forest School of Medicine, Winston-Salem, North Carolina.; c. Department of Radiology, Advanced Neuroscience Imaging Research (ANSIR) Laboratory; University of Texas Southwestern Medical Center, Dallas, Texas.; d. Department of Internal Medicine, Section on Nephrology, Wake Forest School of Medicine, Winston-Salem, North Carolina.

**Keywords:** Blood cell mitochondrial bioenergetics, Type 2 Diabetes (T2D), Brain morphology, Cognitive impairment, Biomarker, Magnetic resonance imaging, African Americans

## Abstract

Blood-based bioenergetic profiling has promising applications as a minimally invasive biomarker of systemic bioenergetic capacity. In this study, we examined peripheral blood mononuclear cell (PBMC) mitochondrial function and brain morphology in a cohort of African Americans with longstanding Type 2 diabetes. Key parameters of PBMC respiration were correlated with white matter, gray matter, and total intracranial volumes. Our analyses indicate that these relationships are primarily driven by the relationship of systemic bioenergetic capacity with total intracranial volume, suggesting that systemic differences in mitochondrial function may play a role in overall brain morphology.

## Introduction

Type 2 diabetes mellitus (T2DM) is one of the most common diseases in older adults (1). Those with T2DM are susceptible to diseases like neuropathy, retinopathy, nephropathy, stroke, and ischemic heart disease, and are more likely to suffer from cognitive impairment and experience a higher risk of dementia (2–4), cerebral infarctions and loss of total gray matter, white matter, and hippocampal volumes (5, 6).

Studies of brain tissues and neurons show that mitochondrial bioenergetics regulate brain energy homeostasis and metabolism during development (7), and affect brain function and cognition (8). Indeed, the brain has an exceptionally high metabolic demand rendering it highly sensitive to changes in systemic bioenergetic capacity (9, 10). There is growing evidence linking central and peripheral metabolic alterations in the pathophysiology of neurodegenerative diseases. Such relationships may be due to intercellular signaling mediated by non-cellular, blood-borne circulating factors such as inflammatory cytokines, redox stress, mitokines, and exosomes (11–15). These factors can have systemic effects on the bioenergetic capacity across multiple organs, as well as circulating cells that are continuously exposed to them. Previous publications from our group support this premise and provide direct evidence that the assessment of mitochondrial function in circulating cells is associated with the bioenergetic capacity of different highly metabolically active organs such as the brain, heart, and skeletal muscle (23, 24). To date, the bioenergetic profiles of peripheral blood mononuclear cells (PBMCs) and platelets have been associated with several age-related disorders, including diabetes, atherosclerosis, and neurodegeneration (16–22).

This study aimed to examine the relationships between the bioenergetic capacity of PBMCs and key features of brain morphology: total gray matter volume (TGM), total white matter volume (TWM), total intracranial volume (TICV). We focused on African American individuals with T2DM who participated in African American-Diabetes Heart Study MIND (AA-DHS MIND). African Americans have a higher risk of T2DM (25), leading to an increased risk for cognitive impairment (26, 27). We tested the hypothesis that PBMC bioenergetics capacity correlates with brain structure and cognitive performance by examining the relationships between PBMC respiration and total and regional brain volumes measured by MRI and Montreal Cognitive Assessment (MoCA) scores. To our knowledge, this is the first study to specifically examine these relationships in an African American population.

## Experimental Methods

### Participants

AA-DHS MIND is a cross-sectional genetic and epidemiologic study designed to evaluate and improve the understanding of risk factors for impaired cognitive performance and to assess cerebral architecture using magnetic resonance imaging (MRI) in African Americans with T2D. It builds on the AA-DHS study, which is an extension of the Diabetes Heart Study (DHS) (28) designed to assess the relationship between cognitive impairment and cerebrovascular disease in an African American cohort. The study was approved by the Wake Forest School of Medicine (WFSM) Institutional Review Board and all participants provided written, informed consent. We recruited 16 unrelated African Americans. Participants (9 women and 7 men) were older (51.7–81.8 years), overweight or obese (body mass index [BMI] > 25.5–50.6 kg/m^2^), and sedentary. Examinations were performed in the WFSM Clinical Research Unit.

### Cerebral Magnetic Resonance Imaging (MRI)

Detailed methods for MRI scans and analyses in AA-DHS MIND have been reported previously (29–31). As previously described, all MRI scans were obtained on a 3T MRI scanner (32). T1-weighted images were analyzed for structural analysis to obtain total intracranial volume, total gray matter and total white matter volumes using the SPM8 segmentation procedure implemented in the VBM8 toolbox (33, 34). Brain MRI was performed at the first visit of the participants.

### Body Weight, Blood Draw and MoCA Examinations

Body weight and fasting blood glucose were assessed at the day of visit for this study. Fasting measures of HbA1c were acquired at the first visit of the participants The Montreal Cognitive Assessment (MoCA), a screening test that assesses cognitive impairment (35), was administered after the participants had breakfast.

### Respirometry of Blood Cells

Mitochondrial oxidative phosphorylation can be measured by evaluating the rate of oxygen consumption in cells and tissues of interest (36–38). Blood cell respirometry was performed using two complementary approaches. Intact PBMCs were assessed with a Seahorse XF24–3 extracellular flux analyzer (Seahorse Bioscience, Billerica, MA). 250,000 PBMCs were loaded into each well and assessed in quadruplicate using previously described methods (39). Briefly, basal oxygen consumption rate (OCR) was monitored prior to chemical additions, followed by OCR measurements after sequential injections of oligomycin (0.75 μM), carbonyl cyanide-4- (trifluoromethoxy) phenylhydrazone (FCCP; 1 μM), and antimycin A + rotenone (A/R; 1 μM each). All chemicals were obtained from SigmaAldrich. PBMC respiration was reported as pmol.min^−1^.

High-resolution respirometry of permeabilized PBMCs was performed in parallel to provide key measures of fatty acid oxidation and respiration driven by individual complexes. For these studies, 4 million PBMCs were loaded into each of 2 chambers of an Oroboros Oxygraph-2K (Oroboros, Innsbruck, Austria). Respirometry was performed following a substrate-uncoupler-inhibitor-titration reference protocol in which multiple substrates and inhibitors are sequentially added to measure oxygen flux due to fatty acid oxidation, followed by oxidative phosphorylation. PBMCs were placed into a chamber with 2 mL mitochondrial respiration medium, MiR05 constituting of 0.5 mM EGTA, 3 mM MgCl_2_, 60 mM lactobionic acid, 20mM taurine, 10mM KH_2_PO_4_, 20mM HEPES, 110 mM D-Sucrose, and 1 g/L fatty acid free BSA, pH 7.1. Chambers were equilibrated at room oxygen concentration at 37°C for at least 30 minutes and routine endogenous respiration was measured, followed by addition of 7.5 mM ADP. Cells were then permeabilized with 0.04 mg/mL digitonin, followed by addition of 0.5 mM octanoylcarnitine to evaluate fatty acid oxidation capacity and 0.05 mM malate to kinetically saturate the fatty acid oxidation pathway. This was followed by sequential addition of 2 mM malate, 10 μM cytochrome c to assess outer mitochondrial membrane integrity, 5 mM pyruvate, 10 mM glutamate, 50 mM succinate, and 10 mM glycerophosphate. These additions target complexes I, II, and ubiquinone or coenzyme Q of the electron transport chain, resulting in detailed measurements of mitochondrial function. The electron transport chain was uncoupled with FCCP (by titrating 0.5 μM FCCP in each step), and then inhibited by addition of complex I and III inhibitors, 0.5 μM rotenone, and 2.5 μM antimycin A, measuring residual oxygen consumption. PBMC respiration was reported as fmol.sec^−1^.cell^−1^.

### Statistical Analyses

Shapiro-Wilk tests were performed to check for normal distribution of all variables. Log transformations were performed for parameters with non-normal distribution. Pearson correlation coefficients were assessed between all variables, both raw and normalized values, and partial correlations adjusted for age and sex were also assessed. Significance was set at an α-level of 0.05. Analyses were performed using SPSS software (SPSS v22; Armonk, NY).

## Results

### Demographic and bioenergetic parameters of the human participants

**Figure 1:**
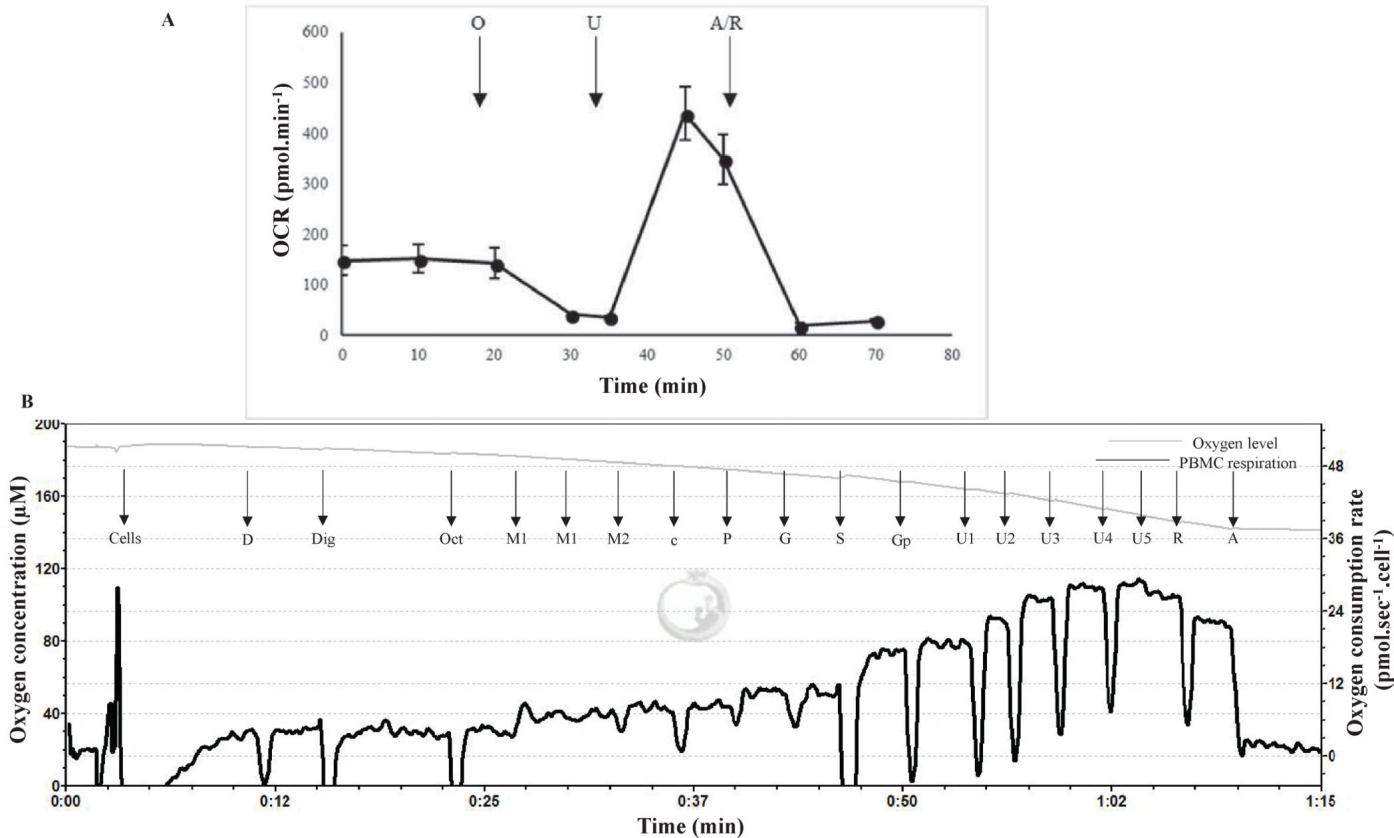
Representative graphs of the two different techniques used to measure PBMC respirometry. Bioenergetic profiles of PBMCs isolated from one participant. Respiration is measured as oxygen consumption rate. **Figure1A:** Representative graph generated by the Seahorse XF24–3 extracellular flux analyzer. **Figure1B:** Representative graph generated by the Oroboros O2K respirometer. As shown in **Figure 1A**, injections were as follows: O=oligomycin, U=uncoupler (FCCP), R=rotenone, A=antimycin A. As shown in **Figure 1B**, multiple substrates and inhibitors were sequentially added to permeabilized cells and measure oxygen flux due to fatty acid oxidation, followed by oxidative phosphorylation. D=adenosine diphosphate, Dig=Digitonin, Oct=Octanoylcarnitine, M1=0.1M malate, M2=0.8M malate, c=cytochrome c, P=pyruvate, G=glutamate, S=succinate, Gp=glycerophosphate, U=uncoupler (FCCP), R=rotenone, A=antimycin A.

Demographic parameters (age, BMI, duration of T2DM, HbAlc), bioenergetic parameters, and brain morphology parameters analyzed are summarized in Table 1. Representative bioenergetic profiles from a participant are shown in Figure 1A **and** 1B.

### Associations between PBMC bioenergetics and brain morphology

Pearson correlation coefficients were used to compare PBMC bioenergetic parameters with brain morphology (Table 2). Representative scatter plots are shown in Supplemental Figures 1A–1D. Basal, maximal FCCP-induced respiration, and ATP-linked respiration of PBMCs significantly positively correlated with total white matter volume (R = 0.666, 0.547, and 0.563), while basal and maximal FCCP-induced respiration correlated significantly with total intracranial volume (0.588 and 0.550). Fatty acid oxidation-mediated oxygen flux (respiration of cells after addition of malate to kinetically saturate the fatty acid oxidation [FAO] pathway) significantly correlated with TWM volume and TICV (R = 0.591 and 0.684). Similar relationships were observed between maximal electron transport (measured by FCCP titration) and TWM volume and TICV. Significant positive association were seen between FAO + complex I activity and TWM volume and TICV. Significant associations were observed between combined FAO + complex I + complex II activity and TWM volume and TICV.

As shown in Table 3, after adjusting for age and sex, basal, maximal FCCP-linked, and ATP-linked bioenergetic capacity of PBMC remained significantly positively correlated with TWM volume and TICV. Spare respiratory capacity showed significant positive correlation with TICV in both cases, and with TGM volume when specifically controlling for sex. Fatty acid oxidation-mediated respiration and FAO + complex I-mediated respiration were significantly positively correlated with TGM volume, TWM volume, and TICV. Similar significant correlations were observed between FAO + complex I + complex II and maximal FCCP-linked respiration and TWM volume and TICV.

As shown in Supplemental tables 1 **through** 4, adjustment for duration of T2DM, BMI and T2DM severity (HbA1c) did not affect these relationships between brain morphology and PBMC bioenergetic capacity.

### Associations between PBMC bioenergetics and normalized brain morphology parameters

Table 4 shows the relationships between PBMC bioenergetics and brain morphologic parameters statistically adjusted for TICV. This adjustment caused all correlations to become less significant, indicating that TICV was the main driver of the associations with TWM volume and TGM volume.

### Associations between PBMC bioenergetic capacity and MoCA test scores

Pearson and partial correlation coefficients were calculated to compare PBMC bioenergetic parameters with MoCA test scores before and after adjusting for age of the participants. The associations are summarized in Table 5. Basal respiration was significantly positively associated with both raw values as well as age adjusted MoCA scores; similar trends were observed for the other bioenergetic parameters.

## Discussion

Mitochondrial bioenergetics plays a key role in the effects of aging on neuronal function (40). Mitochondrial dysfunction is related to numerous age-related diseases, including T2DM, obesity, Parkinson’s, and Alzheimer’s disease (41–44). Recent work from our laboratory and others indicate that measures of mitochondrial function performed in circulating cells can report on systemic bioenergetic capacity and are related to various age-related conditions (45–52). The current study provides the first report that systemic bioenergetic capacity is related to key measures of brain morphology.

Our results indicate that systemic bioenergetic capacity, assessed by PBMC respirometry, is significantly positively related to total intracranial volume (TICV), a parameter estimating maximum pre-morbid brain volume (53). This finding suggests that differences in systemic bioenergetic capacity may be related to the overall development and atrophy of the brain. Our results also indicate that basal respiration of intact PBMCs is significantly positively related to cognitive function, measured using the MoCA assessment. ATP-linked respiration shows a strong trend while other measures in intact PBMCs are not significant. Our results also show that statistically adjusting for age, sex, BMI and T2DM severity (HbA1c) does not affect the relationships observed in this study. It is notable that while BMI and T2DM have been associated with alterations in mitochondrial function in previous studies, the relationship of systemic bioenergetic capacity with brain morphology is independent of these variables.

TICV is currently the most accepted and widely used measure of brain reserve and is associated with higher cognitive performance after adjusting for the amount of pathology in Alzheimer’s disease (15). Moreover, it has been previously reported that greater premorbid brain volume results in better clinical and cognitive outcomes (54, 55). BMI, hyperglycemia and T2DM are associated with brain atrophy, cognitive impairment and dementia, with duration of T2DM strongly associated with brain volume loss (54). This is possibly a result of direct neurologic insult from altered glucose and mitochondrial metabolism, leading to mitochondrial dyshomeostasis and loss of synaptic integrity, affecting brain functions and morphology. Future studies will address whether these central metabolic alterations are relayed to PBMC mitochondria via non-cellular, blood-borne circulating factors that are potentially released from the brain, altering PBMC mitochondrial bioenergetics.

To our knowledge, the assay protocols utilized in this study are the most comprehensive assessment of PBMC bioenergetics to date. We examined the respiration of intact and permeabilized PBMCs in parallel to enable in depth analysis of electron transport chain activity. It should be considered that the measures presented here are interrelated and focused on contributors to overall mitochondrial function. For example, the individual activities of complexes 1 and 2, as well as fatty acid oxidation all contribute to the bioenergetic capacity of a cell. It should be noted that even if we were to choose p=0.01 as the level of significance, key relationships remain significant; particularly the relationships between the basal respiration and the FAO mediated respiration with TWM and TICV. Moreover, these relationships remain when adjusting for age, sex, duration and severity of T2DM, BMI and blood glucose levels.

The composition of the cohort utilized for this study is also unique and representative of a group that is at a greater risk of developing cognitive decline (55). Future studies should be designed to determine if the results can be extended to other cohorts. It is also important to remember that bioenergetic profiling was performed at a single time point. Therefore, longitudinal studies should be designed to more definitely assess the role of PBMC bioenergetic capacity in brain development or degeneration. Blood based bioenergetic profiling is rapidly emerging as a reliable measure of systemic bioenergetic capacity. To date, studies have focused on mixed PBMCs, as performed here, but also other cell types such as platelets and monocytes (52, 56–59). The design of future studies will continue to improve as we continue to advance our understanding of how various circulating cell types reflect bioenergetic changes associated with various conditions and disorders.

## Supplementary Material

1

## Figures and Tables

**Table 1:** Demographics, bioenergetics, and brain morphology parameters.

N=16	Mean	SD	Range
Age (years)	64.42	7.71	51.65 – 81.76
BMI (kg/m^2^)	34.11	7.92	25.51 – 50.64
Duration of T2D (years)	12.97	8.86	3.66 – 39.12
Fasting blood sugar (mg/dL)	150.67	52.53	79 – 283
HbAlc (%)	7.82	1.66	5.8 – 12.6
MoCA score	21.5	4.7	13.00 – 29.00
**Bioenergetic Parameters**			
Basal (pmol.min^−1^)	113.13	51.15	42.05 – 169.35
Maximal uncoupled respiration (pmol.min^−1^)	245.53	139.12	91.30 – 657.92
Spare Respiratory Capacity (pmol.min^−1^)	132.40	94.20	14.30 – 412.14
ATP-linked respiration (pmol.min^−1^)	63.81	45.33	(−19.54) - 111.4
FAO (fmol.sec^−1^.cell^−1^)	2.66 ×10^−3^	1.02 ×10^−3^	1.19 ×10^−3^ - 5.35 ×10^−3^
FAO+ComplexI (fmol.sec^−1^.cell^−1^)	3.66 ×10^−3^	1.86 ×10^−3^	1.175 ×10^−3^ - 8.29 ×10^−3^
FAO+ComplexI+ComplexII (fmol.sec^−1^.cell^−1^)	5.05 ×10^−3^	2.605 ×10^−3^	1.475 ×10^−3^ - 13.09 ×10^−3^
Max ETS (fmol.sec^−1^.cell^−1^)	7.55 ×10^−3^	4.59 ×10^−3^	2.30 ×10^−3^ - 11.70 ×10^−3^
**Brain Anatomy Parameters**			
Total Gray Matter Volume (GM) (cm^3^)	570.85	49.27	492.21 – 648.74
Total White Matter Volume (WM) (cm^3^)	478.73	45.21	422.24 – 553.91
Total Intracranial Volume (TICV) (cm^3^)	1294.10	117.59	1134.77 – 1560.82

Note: PBMC bioenergetic parameters recorded by Seahorse XF24–3 extracellular flux analyzer are reported as oxygen consumption rate (pmol.min^−1^) per 250,000 cells.

**Table 2:** Relationship between PBMC respiration and brain morphology parameters measured by Pearson correlation. Pearson correlation coefficients and p-values for each association are shown. FAO = Fatty Acid Oxidation, ETS = Maximal ETC mediated respiratory system activity. Bold type = p-value ≤ 0.05. Spare respiratory capacity is calculated as the difference between maximal and basal Respiration.

Respirometry Parameters	TGM	TWM	TICV
Basal Respiration	R = 0.338	**R = 0.666**	**R = 0.588**
	p = 0.218	**p = 0.007**	**p = 0.021**
Maximal Respiration	R = 0.375	**R = 0.547**	**R = 0.550**
	p = 0.169	**p = 0.035**	**p = 0.034**
Spare Respiratory Capacity	R = 0.367	R = 0.408	R = 0.477
	p = 0.178	p = 0.131	p = 0.072
ATP-linked Respiration	R = 0.253	**R = 0.563**	R = 0.490
	p = 0.364	**p = 0.029**	p = 0.064
FAO	R = 0.477	**R = 0.591**	**R = 0.684**
	p = 0.062	**p = 0.016**	**p = 0.003**
FAO+ComplexI	R = 0.467	**R = 0.519**	**R = 0.564**
	p = 0.068	**p = 0.040**	**p = 0.023**
FAO+Compl exI+ComplexII	R = 0.375	**R = 0.502**	**R = 0.528**
	p = 0.152	**p = 0.047**	**p = 0.035**
Max ETS	R = 0.349	**R = 0.503**	**R = 0.503**
	p = 0.199	**p = 0.047**	**p = 0.047**

**Table 3:** Relationship between PBMC respiration and brain morphologic parameters statistically adjusted for age and sex. Correlation coefficients and p-values for each association are shown. Bold type = p-value ≤ 0.05.

	Adjusted for Age			Adjusted for Sex		
Respirometry Parameters	TGM	TWM	TICV	TGM	TWM	TICV
Basal Respiration	R = 0.346	**R = 0.668**	**R = 0.607**	**R = 0.570**	**R = 0.705**	**R = 0.709**
	p = 0.225	**p = 0.009**	**p = 0.021**	**p = 0.033**	**p = 0.005**	**p = 0.005**
Maximal Respiration	R = 0.408	**R = 0.566**	**R = 0.610**	**R = 0.627**	**R = 0.586**	**R = 0.676**
	p = 0.148	**p = 0.035**	**p = 0.021**	**p = 0.016**	**p = 0.028**	**p = 0.008**
Spare Respiratory Capacity	R = 0.412	R = 0.430	**R = 0.552**	R = 0.489	R = 0.416	**R = 0.525**
	p = 0.143	p = 0.125	**p = 0.041**	p = 0.076	p = 0.139	**p = 0.054**
ATP-linked Respiration	R = 0.258	**R = 0.564**	R = 0.504	**R = 0.532**	**R = 0.620**	**R = 0.644**
	p = 0.372	**p = 0.036**	p = 0.066	**p = 0.050**	**p = 0.018**	**p = 0.013**
FAO	R = 0.474	**R = 0.589**	**R = 0.683**	R = 0.460	**R = 0.603**	**R = 0.674**
	p = 0.074	**p = 0.021**	**p = 0.005**	p = 0.084	**p = 0.017**	**p = 0.006**
FAO+ComplexI	R = 0.483	**R = 0.529**	**R = 0.594**	R = 0.436	**R = 0.534**	**R = 0.547**
	p = 0.068	**p = 0.043**	**p = 0.020**	p = 0.105	**p = 0.040**	**p = 0.035**
FAO+ComplexI+ComplexII	R = 0.411	**R = 0.531**	**R = 0.593**	R = 0.443	**R = 0.502**	**R = 0.548**
	p = 0.128	**p = 0.042**	**p = 0.020**	p = 0.099	**p = 0.057**	**p = 0.034**
Max ETS	R = 0.379	**R = 0.527**	**R = 0.556**	R = 0.393	**R = 0.503**	**R = 0.513**
	p = 0.164	**p = 0.044**	**p = 0.031**	p = 0.147	**p = 0.056**	**p = 0.050**

**Table 4:** Relationships between PBMC respiration and brain morphologic parameters statistically adjusted for TICV measured by Partial correlation. Correlation coefficients and p-values for each association are shown.

	Adjusted for TICV	
Respirometry Parameters	TGM	TWM
Basal Respiration	R = −0.105	R = 0.510
	p = 0.734	p = 0.062
Maximal Respiration	R = 0.051	R = 0.313
	p = 0.870	p = 0.277
Spare Respiratory Capacity	R = 0.165	R = 0.143
	p = 0.589	p = 0.627
ATP-linked Respiration	R = −0.144	R = 0.445
	p = 0.638	p = 0.110
FAO	R = 0.089	R = 0.251
	p = 0.761	p = 0.366
FAO+ComplexI	R = 0.166	R = 0.282
	p = 0.571	p = 0.308
FAO+ComplexI+ComplexII	R = −0.003	R = 0.322
	p = 0.991	p = 0.242
Max ETS	R = 0.030	R = 0.386
	p = 0.918	p = 0.156

**Table 5:** Relationship between PBMC respiration and MoCA scores (raw values and values adjusted for age) measured by Pearson correlation (raw values) (first panel) and partial correlation (adjusted for age) (second panel). Pearson correlation coefficients and p-values for each association are shown. Bold type = p-value ≤ 0.05.

	Raw values	Adjusted for age
Respirometry Parameters	MoCA	MoCA
Basal Respiration	**R = 0.571**	**R = 0.579**
	**p = 0.026**	**p = 0.030**
Maximal Respiration	R = 0.301	R = 0.328
	p = 0.276	p = 0.252
Spare Respiratory Capacity	R = −0.067	R = −0.050
	p = 0.812	p = 0.866
ATP-linked Respiration	R = 0.479	R = 0.484
	p = 0.071	p = 0.079
FAO	R = 0.196	R = 0.191
	p = 0.468	p = 0.495
FAO+ComplexI	R = −0.002	R = 0.006
	p = 0.995	p = 0.982
FAO+Compl exI+ComplexII	R = 0.131	R = 0.153
	p = 0.630	p = 0.586
Max ETS	R = 0.155	R = 0.175
	p = 0.566	p = 0.534

## Clinical Perspectives

- African American individuals represent a cohort experiencing a higher risk of T2DM, consequently exhibiting an increased risk of cognitive decline.
- PBMC bioenergetic capacity is directly related to overall brain morphology and cognitive function.
- Blood-based bioenergetic profiling is a promising minimally invasive biomarker of systemic bioenergetic capacity.

## Abbreviations list

T2DM: Type 2 Diabetes Mellitus
PBMC: Peripheral blood mononuclear cell
AA-DHS Mind: African American - Diabetic Heart Study Mind
MRI: Magnetic Resonance Imaging
3T MRI: 3 Tesla Magnetic Resonance Imaging
MoCA: Montreal Cognitive Assessment
WFSM: Wake Forest School of Medicine
BMI: Basal Metabolic Rate
OCR: Oxygen Consumption Rate
FAO: Fatty Acid Oxidation
ETS: Maximal ETC mediated respiratory system activity
FCCP: Carbonyl cyanide-4- (trifluoromethoxy) phenylhydrazone
MiR05: Mitochondrial Respirometry Solution
HEPES: 4-(2-hydroxyethyl)-1-piperazineethanesulfonic acid
BSA: Bovine Serum Albumin
SPSS: Statistical Package for the Social Sciences
