# Supplementary material for: Blood-based bioenergetic profiling is related to differences in brain morphology in African Americans with Type 2 diabetes
Source: Clin Sci (Lond). Author manuscript; Available in PMC 2019 May 13. (PMC6512318; doi:10.1042/CS20180690)

Supplemental Figure 1A-1D: Scatter plots of associations between PBMC respiration and total white matter and total intracranial volume measurements. Total white matter volume and total intracranial volumes (cm^3) are plotted against the different respiratory parameters tested. Pearson correlation coefficients and p-values for each association are shown.

Supplemental Table 1

	Adjusted for Duration of T2DM			
**Respirometry Parameters**	**TGM**	**TWM**	**TICV**	**MoCA**
Basal Respiration	R = 0.337	**R = 0.704**	**R = 0.588**	**R = 0.572**
	p = 0.238	**p = 0.005**	**p = 0.027**	**p = 0.033**
Maximal Respiration	R = 0.373	**R = 0.589**	**R = 0.550**	R = 0.296
	p = 0.189	**p = 0.027**	**p = 0.042**	p = 0.304
Spare Respiratory Capacity	R = 0.368	R = 0.421	R = 0.477	R = -0.066
	p = 0.195	p = 0.134	p = 0.084	p = 0.823
ATP-linked Respiration	R = 0.250	**R = 0.652**	R = 0.494	R = 0.466
	p = 0.389	**p = 0.012**	p = 0.073	p = 0.093
FAO	R = 0.471	**R = 0.606**	**R = 0.684**	R = 0.204
	p = 0.071	**p = 0.017**	**p = 0.005**	p = 0.466
FAO+ComplexI	R = 0.481	R = 0.484	**R = 0.574**	R = 0.026
	p = 0.070	p = 0.068	**p = 0.025**	p = 0.928
FAO+ComplexI+ComplexII	R = 0.382	R = 0.480	**R = 0.532**	R = 0.154
	p = 0.161	p = 0.070	**p = 0.041**	p = 0.584
Max ETS	R = 0.361	R = 0.463	**R = 0.512**	R = 0.190
	p = 0.186	p = 0.082	**p = 0.051**	p = 0.497

Supplemental Table 2

	**Adjusted for BMI**			
**Respirometry Parameters**	**TGM**	**TWM**	**TICV**	**MoCA**
Basal Respiration	R = 0.297	**R = 0.653**	**R = 0.563**	**R = 0.538**
	p = 0.325	**p = 0.016**	**p = 0.045**	**p = 0.058**
Maximal Respiration	R = 0.359	**R = 0.563**	**R = 0.561**	R = 0.283
	p = 0.228	**p = 0.045**	**p = 0.046**	p = 0.349
Spare Respiratory Capacity	R = 0.427	R = 0.507	**R = 0.582**	R = -0.006
	p = 0.146	p = 0.077	**p = 0.037**	p = 0.984
ATP-linked Respiration	R = 0.197	R = 0.526	R = 0.441	R = 0.420
	p = 0.519	p = 0.065	p = 0.131	p = 0.153
FAO	R = 0.496	**R = 0.653**	**R = 0.748**	R = 0.213
	p = 0.071	**p = 0.011**	**p = 0.002**	p = 0.465
FAO+ComplexI	**R = 0.535**	**R = 0.634**	**R = 0.681**	R = 0.063
	**p = 0.049**	**p = 0.015**	**p = 0.007**	p = 0.830
FAO+ComplexI+ComplexII	R = 0.415	**R = 0.598**	**R = 0.620**	R = 0.179
	p = 0.140	**p = 0.024**	**p = 0.018**	p = 0.541
Max ETS	R = 0.428	**R = 0.651**	**R = 0.647**	R = 0.260
	p = 0.127	**p = 0.012**	**p = 0.012**	p = 0.370

Supplemental Table 3

	Adjusted for HbA1c			Adjusted for Blood Glucose		
**Respirometry Parameters**	**TGM**	**TWM**	**TICV**	**TGM**	**TWM**	**TICV**
Basal Respiration	R = 0.368	**R = 0.772**	**R = 0.634**	R = 0.347	**R = 0.677**	**R = 0.572**
	p = 0.195	**p = 0.001**	**p = 0.015**	p = 0.224	**p = 0.008**	**p = 0.032**
Maximal Respiration	R = 0.443	**R = 0.727**	**R = 0.648**	R = 0.375	**R = 0.542**	**R = 0.535**
	p = 0.112	**p = 0.003**	**p = 0.012**	p = 0.187	**p = 0.046**	**p = 0.049**
Spare Respiratory Capacity	R = 0.443	**R = 0.586**	**R = 0.579**	R = 0.366	R = 0.403	R = 0.471
	p = 0.113	**p = 0.028**	**p = 0.030**	p = 0.198	p = 0.153	p = 0.089
ATP-linked Respiration	R = 0.300	**R = 0.712**	**R = 0.562**	R = 0.252	**R = 0.560**	R = 0.468
	p = 0.297	**p = 0.004**	**p = 0.036**	p = 0.385	**p = 0.037**	p = 0.091
FAO	**R = 0.513**	**R = 0.703**	**R = 0.740**	R = 0.484	**R = 0.588**	**R = 0.671**
	**p = 0.050**	**p = 0.003**	**p = 0.002**	p = 0.068	**p = 0.021**	**p = 0.006**
FAO+ComplexI	R = 0.501	**R = 0.621**	**R = 0.614**	R = 0.466	**R = 0.513**	**R = 0.557**
	p = 0.057	**p = 0.013**	**p = 0.015**	p = 0.080	**p = 0.050**	**p = 0.031**
FAO+ComplexI+ComplexII	R = 0.445	**R = 0.685**	**R = 0.630**	R = 0.375	R = 0.494	R = 0.509
	p = 0.096	**p = 0.005**	**p = 0.012**	p = 0.168	p = 0.061	p = 0.052
Max ETS	R = 0.419	**R = 0.689**	**R = 0.604**	R = 0.349	R = 0.495	R = 0.483
	p = 0.120	**p = 0.005**	**p = 0.017**	p = 0.203	p = 0.061	p = 0.068

Supplemental Table 4

	**Adjusted for HbA1c**	**Adjusted for Blood Glucose**
**Respirometry Parameters**	**MoCA**	**MoCA**
Basal Respiration	**R = 0.568**	R = 0.488
	**p = 0.034**	p = 0.076
Maximal Respiration	R = 0.293	R = 0.235
	p = 0.309	p = 0.419
Spare Respiratory Capacity	R = -0.099	R = -0.168
	p = 0.735	p = 0.565
ATP-linked Respiration	R = 0.477	R = 0.421
	p = 0.084	p = 0.134
FAO	R = 0.190	R = 0.021
	p = 0.497	p = 0.940
FAO+ComplexI	R = -0.009	R = -0.100
	p = 0.974	p = 0.724
FAO+ComplexI+ComplexII	R = 0.120	R = -0.029
	p = 0.669	p = 0.918
Max ETS	R = 0.146	R = 0.009
	p = 0.604	p = 0.974

Supplemental Figure 1A

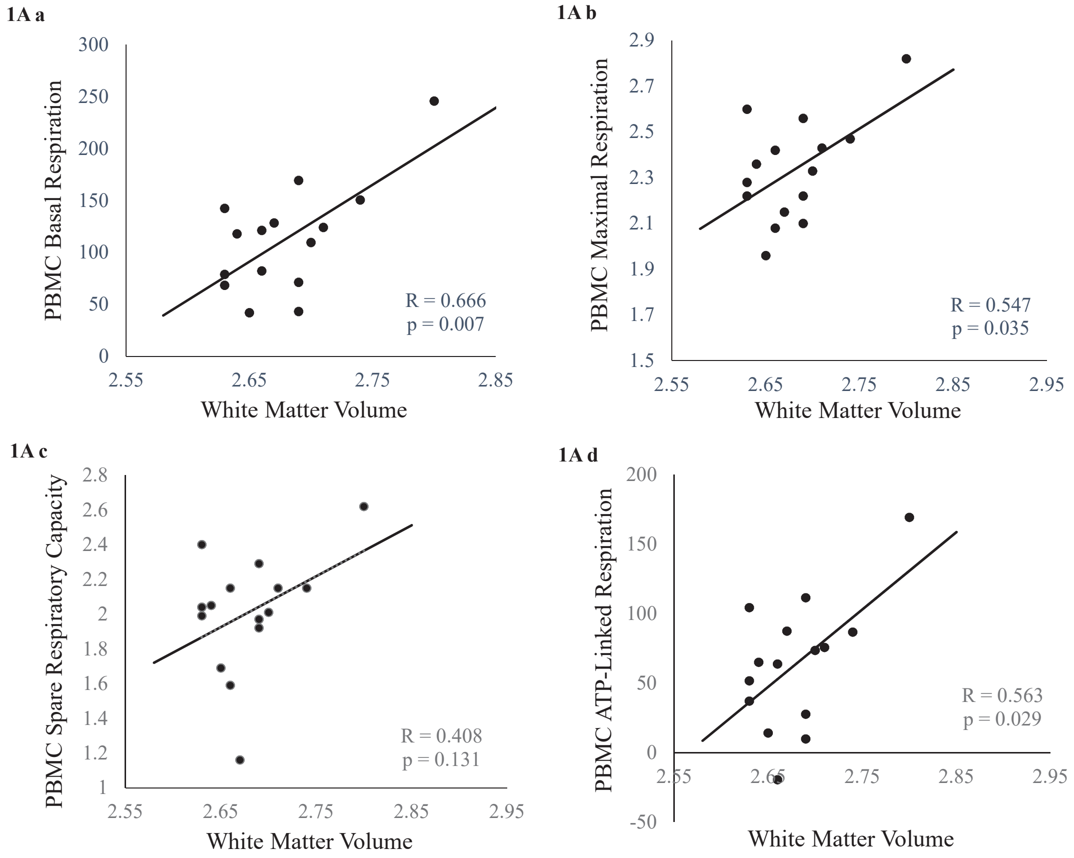

Supplemental Figure 1B

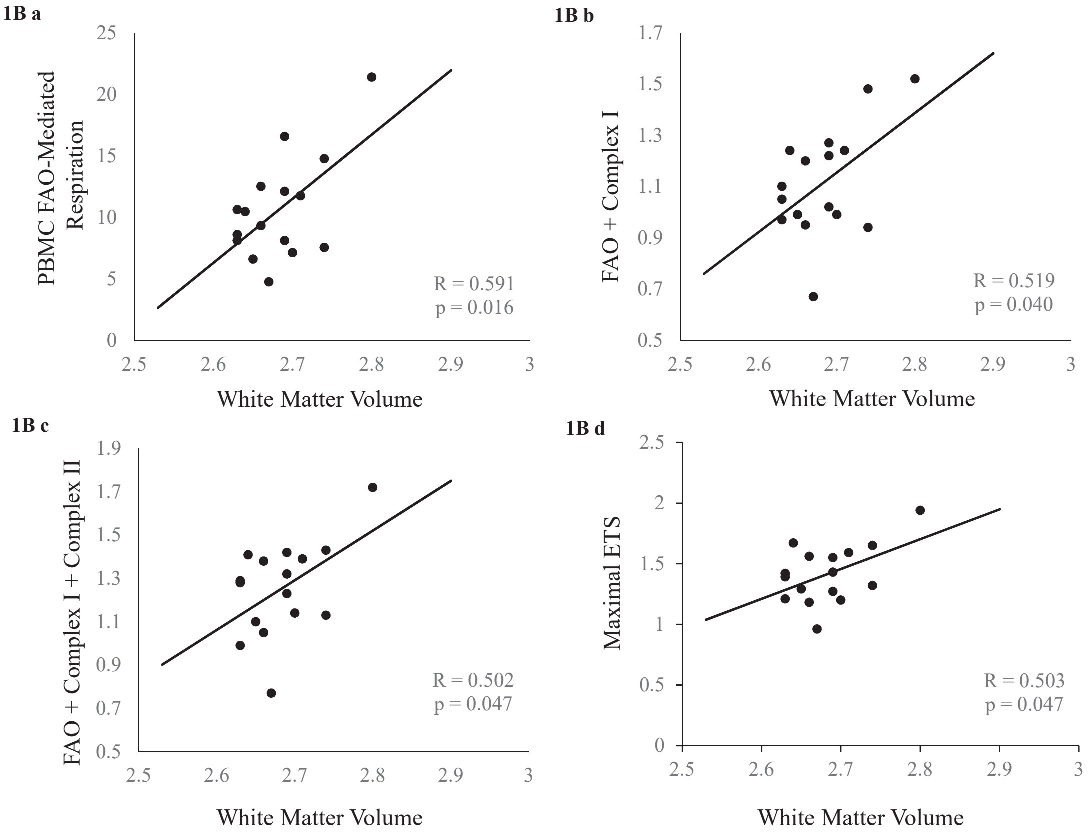

Supplemental Figure 1C

1C a

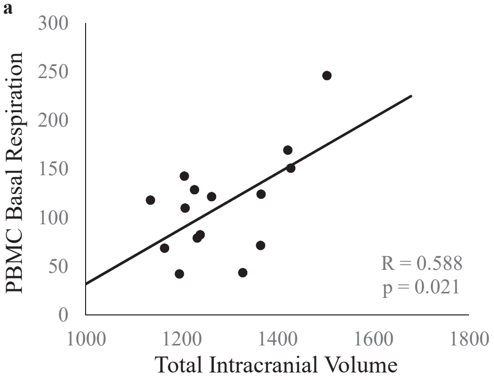

1C b

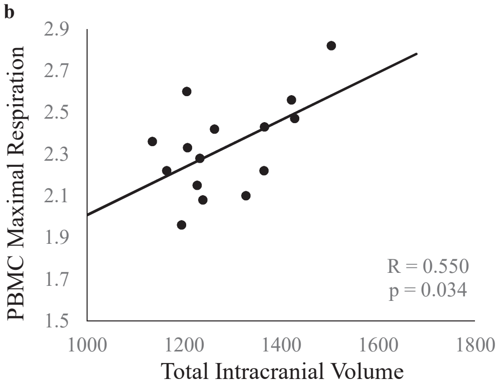

1C c

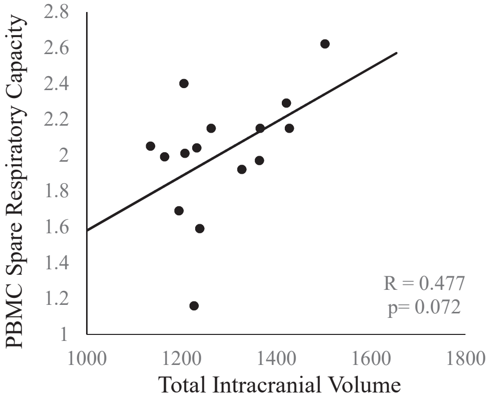

1C d

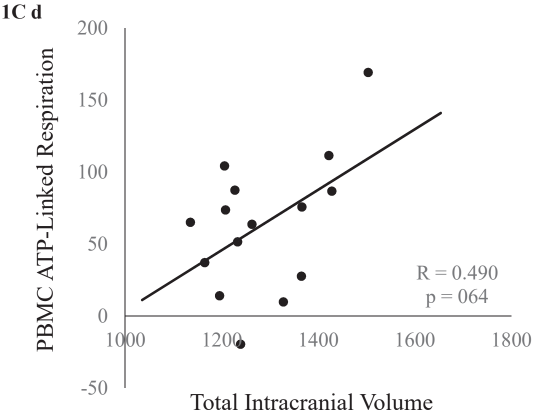

Supplemental Figure 1D

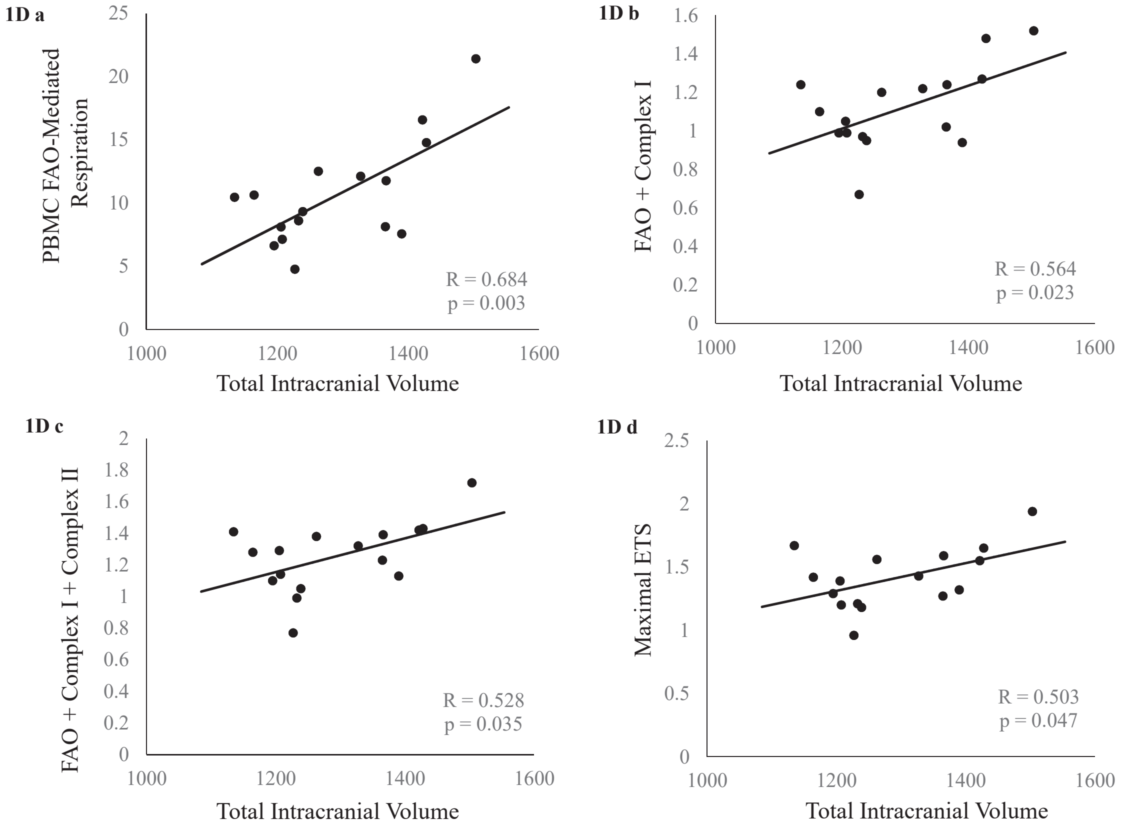

Supplement: 1 [file NIHMS1019126-supplement-1.pdf]
